# Editorial: Combination Therapy for Hypothyroidism: The Journey From Bench to Bedside

**DOI:** 10.3389/fendo.2020.00422

**Published:** 2020-06-25

**Authors:** Jacqueline Jonklaas, Anne R. Cappola, Francesco S. Celi

**Affiliations:** ^1^Division of Endocrinology, Georgetown University, Washington, DC, United States; ^2^Division of Endocrinology, Diabetes, and Metabolism, Perelman School of Medicine, University of Pennsylvania, Philadelphia, PA, United States; ^3^Division of Endocrinology, Virginia Commonwealth University, Richmond, VA, United States

**Keywords:** hypothyroidism, levothyroxine, combination therapy, patient outcomes, clinical trials, basic and translational research

The debate about the optimum replacement therapy for hypothyroidism has been active for many years, and continues to be an area of interest for basic and clinical researchers, practicing clinicians, and patients. In this Research Topic “combination therapy for hypothyroidism,” we bring together a collection of 16 diverse articles describing basic research, clinical research, hypotheses, and opinion pieces that are woven together to illustrate the rich tapestry of this topic.

The importance of thyroid hormone for optimal function of many different organ systems is well-documented. Within the articles included in this topic are three articles exploring the importance of thyroid hormone for cardiometabolic function. One article by Forini et al. discusses whether disordered epigenetic remodeling of chromatin structure and interplay with non-coding RNA may contribute to the cardiac dysfunction seen in low triiodothyronine (T3) states and discusses how the appropriately timed reversal of the low T3 state may improve cardiac function. Another article by Mastorci et al. also reviews the cardioprotective effects of T3, including pathways involving the non-genomic effects of T3. A third article by Stamatouli et al. reviews the effect of thyroid hormone deficiency on diverse cardiometabolic clinical indices such as impaired lipid profile, chronic inflammation, increased oxidative stress, and increased insulin resistance.

The importance of individualized therapy for hypothyroidism is accented in an analysis by Hoermann et al.. By analyzing the thyroid analytes in patients being treated with levothyroxine, intra-individual clustering of thyroid parameters was revealed, although such clustering tended to be masked when data from many individuals were averaged, perhaps leading to consideration of personalized treatment goals during therapy. A treatise by Köhrle et al. discusses measurement of the thyroid hormone metabolite 3,5 diiothyronine. These authors describe altered concentration of 3,5 diiothyronine with certain chronic illnesses. They speculate as to whether altered concentrations of 3,5 diiothyronine could contribute to dissatisfaction with levothyroxine monotherapy. Measurement of another thyroid hormone metabolite, reverse T3, has always been a controversial topic and has been of interest to subsets of clinicians and patients. Gomes-Lima et al. review the small amount of literature addressing this topic and conclude that there is insufficient evidence to suggest that measurement of reverse T3 might be useful for guiding combination therapy.

An investigation reported by Jonklaas et al. based on a survey of American Thyroid Association members illustrates that not only do physicians prescribe combination therapy for their patients, but that also they are prescribing more combination therapy over time. A review by McAninch and Bianco provides some history of the use of thyroid hormone preparations. Their discussion includes the potential personalization of thyroid hormone treatment based on an individual's type 2 deiodinase genotype and the possibility that individuals homozygous for the Thr92Ala polymorphism may have impaired thyroid signaling.

Patient reported outcomes and patient preferences are important when assessing the individual patient's response to their hypothyroidism therapy, and these parameters will no doubt be key outcomes in any future combination therapy trials. One patient-reported outcome discussed in this topic collection is a novel hypothyroidism symptoms scale described and tested by Brokhin et al.. A meta-analysis by Akirov et al. shows that patient preference for combination therapy does not differ from that which would be expected by chance, thus illustrating that careful design of future combination therapy trials is essential to fully explore patient preferences.

A review provided by Taylor et al. explores dissatisfaction with levothyroxine therapy, and discusses both the current approach that could be taken to therapeutic trials of liothyronine in individual patients and also potential biomarkers that could be utilized to guide use of combination therapy. They also touch upon the factors that would be important to consider in any future trials of combination therapy. Using computer modeling, another analysis by DiStefano and Jonklaas examines the residual endogenous thyroid function of individuals being treated with thyroid hormone and concludes that the varying degrees of residual thyroid function retained by patients may be one of the variables contributing to the heterogeneous results obtained from the published studies of combination therapy.

Two complementary articles by Cappola and Madan and Celi et al. address our current equipoise with respect to trials of combination therapy and outline the attributes that the authors believe are critical to avoid the shortcomings of previously published combination therapy trials, and to ensure the success of future trials. In addition to the need for randomization, blinding, and placebo-control, both these articles stress the importance of study population, dosing strategy, appropriate selection of primary and secondary outcomes, and adequate statistical power.

Two very different approaches to attempting to develop a sustained release triiodothyronine (T3) or liothyronine (LT3) preparation are illustrated in the review by Idrees et al. and the original research article by Santini et al.. In the former the authors describe a number of products, including a poly-zinc-LT3 formulation that has been tested in rats and may be entering phase I trials in humans within a few years (Idrees et al.), while in the latter the investigators show that stable serum levels of T3 can be achieved by administration of the non-deiodinative T3 metabolite, T3-sulfate (Santini et al.).

In summary, the articles in this collection examine tissue-specific aspects thyroid hormone action, potential laboratory markers of thyroid hormone action, personalization of thyroid hormone therapy, patient-reported outcomes that include patient satisfaction, and physician practices. They also explore potential design of future combination therapy trials and the possibility of a sustained release T3 preparation that may allow fulfillment of the promise of physiologic dosing of combination therapy. We hope that these articles will help clinicians consider whether they wish to offer combination therapy to their patients, and what parameters they would follow to gauge the success of the therapy. We also hope that this collection of articles will provide a framework for researchers designing future trials of combination therapy to determine if there are indeed patients for whom combination therapy is a more satisfactory treatment.

## Author Contributions

JJ, AC, and FC each contributed to the conception and manuscript preparation for this editorial. All authors contributed to the article and approved the submitted version.

## Conflict of Interest

FC was a consultant for IBSA and Acella. The remaining authors declare that the research was conducted in the absence of any commercial or financial relationships that could be construed as a potential conflict of interest.

